# Standardization of the Computerized Battery for Neuropsychological Evaluation of Children (BENCI) in an urban setting, in Kenya: a study protocol

**DOI:** 10.1186/s13104-019-4830-y

**Published:** 2019-12-09

**Authors:** Rachel Wanjiru Maina, Amina Abubakar, Perez-Garcia Miguel, Fons J. R. Van De Vijver, Manasi Kumar

**Affiliations:** 1 Department of Clinical Medicine and Therapuetics, University of Nairobi, Nairobi, 10834-00400 Kenya; 2 Department of Culture Studies, Tilburg University, Tilburg, The Netherlands; 3 Neurosciences Unit, KEMRI-Wellcome Trust Research Programme, Kilifi, Kenya; 4 Institute for Human Development, Aga Khan University, Nairobi, Kenya; 5 Mind, Brain and Behavior Research Center (CIMCYC), University of Granada, Granada, Spain; 6 Department of Psychology, Higher School of Economics, Ulitsa, Russia; 7 Department of Psychiatry, University of Nairobi, Nairobi, Kenya

**Keywords:** Cross-cultural neuropsychological assessment, Cognitive functioning in Kenyan children, Reliability, Convergent validity, Construct validity, Discriminant validity and neurocognitive tests

## Abstract

**Objective:**

In sub Saharan Africa one of the key challenges in assessment using neuropsychological tools has been the lack of adequately validated and easily implementable measures. This study will translate into English, adapt and standardize the Computerized Battery for Neuropsychological Evaluation of Children (BENCI). The BENCI battery will be adapted using back-translation design, comprehensive cultural adaptation and standardized in a case–control study involving two groups of children: HIV infected and HIV unexposed, uninfected children. The content adaptation will be iteratively carried out using knowledge of English and feedback from pilot testing with children. The proposed study will first involve the cultural adaptation of the BENCI. It will then recruit 544 children aged 8–11 years with half of them being HIV+, while the other half will be HIV unexposed-uninfected. Test–retest reliability will be analyzed using Pearson’s correlation while ANOVA and correlational analyses will be used to calculate discriminant, convergent and construct validity.

**Results:**

This study will result in an open access adequately adapted and standardized measure of neuropsychological functioning for use with children in East Africa. The protocol paper provides an opportunity to share the planned methods and approaches.

## Introduction

Children growing up in low and middle-income countries (LAMICs) are at a significant risk of experiencing neurocognitive impairment due to exposure to multiple risk factors [1]. However, the true burden of neurocognitive impairment is not documented largely due to a shortage of adequately standardized, easy to implement measures within these contexts [2, 3]. While a host of neuropsychological tools are currently available, unfortunately, they are not widely tested in African settings due to resource limitations. There is evidence to indicate that importing western measures into LAMICs without adequate attention to adaptation, standardization and validation can contribute to significant challenges in the validity of the data.

Recent efforts indicate that using systematic adaptation process can contribute to the development of neuropsychological measures that can be adequately used in LAMICs settings [4]. We would like to contribute to the body of literature by translating, adapting and providing validity data for the English version of The Computerized Battery for Neuropsychological Evaluation of Children (BENCI). BENCI is a neuropsychological tool originally developed and standardized in Spanish. It aims to capture diverse cognitive functions [5]. Some of the key neuropsychological domains assessed by BENCI include executive functioning, attention, processing speed, language, visual and verbal memory, and visuo-motor coordination.

One attractive feature of the BENCI is its computerized nature which makes it relatively easy to administer and get results readily and paper-free. The BENCI has good psychometric properties in terms of validity and reliability [5]. It has demonstrated good discriminant and construct validity, as well as test retest reliability in Morocco [5] and Ecuador [6]. The battery is relatively easy to administer as there are trial tests in between the subtests. These properties of the BENCI make it favorable for adaptation and standardization in the Kenyan children population. Earlier studies such as those by Holding and colleagues indicated there was a need to have sufficient trials for children in our settings [7]. In addition, BENCI is attractive since it is an open access tool for all mental health professionals particularly those who work in LAMIC will find it very beneficial as it was developed specifically for such contexts and there is no fee tied to its use.

The general objective of this study is to establish the reliability and validity of the BENCI and its utility in monitoring outcomes among HIV positive school going children. The specific objectives are:

- To evaluate the internal consistency, and re-test reliability of the BENCI;
- To evaluate the construct and criterion validity of these measures;
- To evaluate discriminative validity of the measures by comparing the performance of HIV infected and HIV exposed uninfected school going children.

## Main text

### Methods

#### Design

A three-phased approach will be carried out. In the first phase, the linguistic and semantic equivalence of the BENCI content will be ensured through back translation design from Spanish to English by two translators. An evaluation of the tools structure and appropriateness will also be done where psychologists will check for the appropriateness of the pictures and other materials. In the second phase, a pilot study among 10 children will evaluate the appropriateness of the items including pictures and instructions. While in the third phase, the psychometric properties of the English version of the BENCI with regards to the Kenyan population of HIV+ children will be evaluated in a case control study at a HIV programme and three public schools.

#### Study settings

One of the study sites will be a county level HIV programme, which is an outpatient programme catering for HIV infected individuals and their families from culturally diverse backgrounds within resource poor settings.

The normative data will be collected from 3 primary schools in the resource poor setting in Nairobi County.

The two settings are in the Kenyan capital city of Nairobi which has a high level of literacy (87.1%) with English being the primary language of instruction in the schools [8]. An English version of the BENCI is therefore ideal for adaptation. We will ensure that the level of English being used is basic and understand to the target population by pilot testing.

#### Instruments description

A socio-demographic questionnaire will incorporate elements such as age, gender and education background among other variables.

**Fig. 1:**
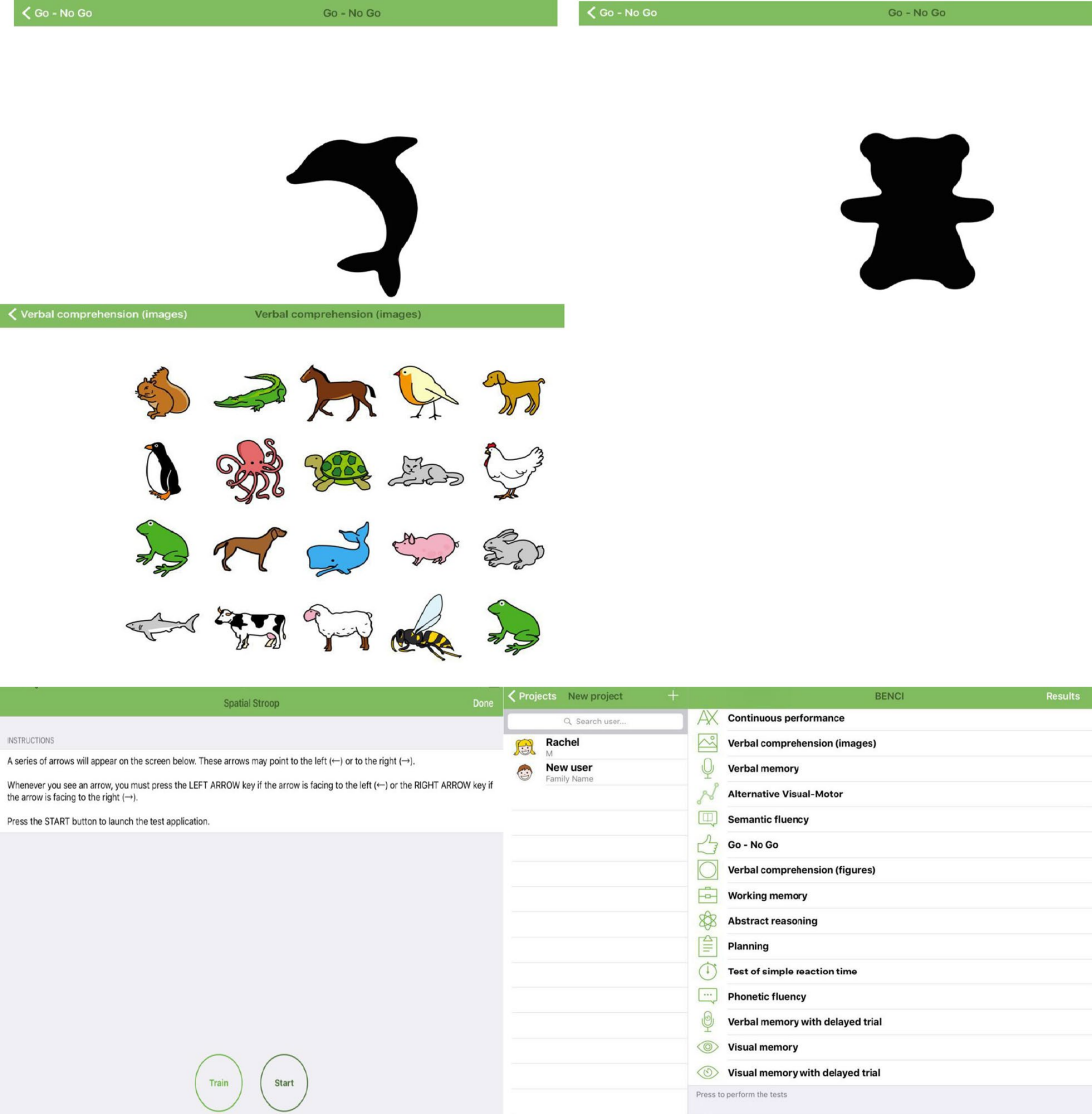
Sub-tests screenshots

A breakdown of measures within each cognitive domain within BENCI is available in Table 1. The BENCI battery was developed using neuropsychological procedures that are valid with regards to the neuropsychological assessment literature which includes DSM V preferred domain assessments [9]. The test has norms for children aged 6–11 years. The test can be administered within 75 min with a 10-min break in between the 14 neuropsychological tests as shown in Fig. 1.

**Table 1:** Comparison of domains and tests used in each domain

BENCI (90 min)		Kilifi Toolkit (120 min)	
Domain	Test	Domain	Test
Processing speed	Simple reaction time test (a plus sign of the screen prompts the child to press a key on the keyboard fast)	–	–
Visuo-motor coordination	Visuo-motor test (involves connection of elements/number in a given sequence)	Verbal/visual selective reminding	Self-ordered pointing test—SOPT (Selection of pictures displayed in varying positions on separate sheets in sets of 6, 8, 10, and 12. As each page is turned the subject is required to identify all members of the set, but to point to each item of the set only once. Touching a picture more than once is considered an error)
Sustained attention	Continuous performance test (respondent presses any key every time the required stimulus appears)	Auditory sustained and selective attention Visual sustained and selective attention	Score (the subject is required to place beads on one of two plates only after a special sound is heard on a cassette tape) People search (a stimulus sheet comprising complete and incomplete stick figures is presented. The subject is required to cross out only complete figures, as quickly as possible)
Memory	Verbal memory test (child listens to some words then repeats the ones remembered) Verbal memory delayed recall test (the series of words said are repeated after 20 min) Verbal memory essay of Recognition test (words are read out loud and respondents identifies those that were in the previous list) Visual memory (series of images are presented after which respondents verbalizes those remembered) Visual memory delayed essay (the images remembered are said out loud after 20 min) Visual memory essay of recognition (respondent identifies if images presented were in previous list)	Memory	Verbal memory: verbal list learning—VLL (two lists of 15 items are read out to the child as a shopping list. The first is presented five times and the second only once)
Language	Verbal comprehension images test (respondent matches images to given conditions) Verbal comprehension figures (respondent matches geographic shapes to given conditions) Phonetic fluency (a letter is presented and respondents verbalizes all words that start with the letter given)	–	–
Executive functioning	Working memory (a list of color and numbers are said and respondent repeats the numbers then the colors) Abstract reasoning (respondent completes a logical series by selecting the right element) Semantic fluency (a category is given and respondents says the elements known in that category) Inhibition: Go/NO-GO (respondents identifies distinguishing factor between two elements and later identify the distinguishing element) Flexibility: spatial stroop (respondent matches arrow directions to arrow labels) (two components of spatial stroop—attention shifting task measures flexibility while proper spatial stroop task measures inhibition) Flexibility: alternate visuo-motor (is flexibility measure that involves two distinct series in which the respondent should connect alternatively) Planning: attraction park (respondent chooses a number of attractions according to money in hand with each attraction chosen expiring after a given period)	Executive functioning	Working memory: dots (a sheet of eight designs made of dots. The examiner identifies the “special” dot in each design, from a series of one up to eight. The subject is required to point at the special dot. Three trials are administered at each level as required) Reasoning: colored progressive matrices—CPM (three sets with 12 matrices made of abstract patterns. The subject is asked to complete the matrix by placing one of a choice of four patterns in the empty space) Attention and attention shift: contingency naming test—CNT (the child is taught a series of rules to name nine drawings displayed in a single series. Each drawing consists of a large outer colored shape and a smaller inner colored shape. Each drawing is named according to the shape or color of one of its two shapes. The rules taught for selecting the name of the item become more complex over four trials) Planning: tower test (three colored wooden balls are moved between three pegs to match a goal position. Time and number of moves required are recorded)

The units of measurement in the BENCI and Kilifi Toolkit are reaction time (RA), total number of correct answers (CA) and number of moves. Measurement in KABC II is through the Fluid Crystallized Index

The Kilifi Neuropsychological Tool Kit comprises of a set of measures adapted and standardized from published measures. The tools have good psychometric properties with split half reliability being between .70 and .84 while internal consistency was ≥ .70 [10]. Some of the cognitive domains they cover include executive functioning, memory and attention. The reliability of the assessment tools is tested among children aged 8–11 years; hence the tools have norms befitting this age group. The measures are all paper and pencil based. These measures are administered to allow for comparison in performance in a paper–pencil test vs. a computerized battery. The tools can be administered within 120 min. See Table 1 for the assessment tools within the two tests.

#### Procedure for translation measures and data collection

The Guidelines for Translating and Adapting Tests developed by the International Test Commission will be used in adapting and standardizing the tool [11]. Permission will first be sought from the developers of the BENCI for modification of the tool. The test developers will also be involved in the modification in ensuring the modifications suggested do not in compromise the validity of the tool. Also, in ensuring the changes are integrated in the computerized version of the tool. Since the BENCI is originally in Spanish, two bilingual translators, one whose native language is Spanish and the other whose native language is English, will be involved in translating the battery. One will translate the tools from Spanish to English and the other check for linguistic and cultural consistency of the English version. Clinical psychologists and psychiatrists, who will be part of the data collection team, will form the third team that will check for synthesis. At this stage the culturally adapted version will be assessed and evaluated against the tool’s original markers. This is where cultural references in the Spanish version that are unfamiliar within the Kenyan children setting will be identified and identical but familiar references integrated. The tool’s structure and appropriateness (such as grammatical correctness) will then be evaluated. A word for word literal translation from Spanish to English may result in grammar and format errors. To curtail such structural problems, the translators as well as the investigators will check the appropriateness of the translated version while maintaining the characteristics of the original test. We will aim for conceptual translations as opposed to literal translations.

A pretest of the BENCI will be carried out in order to identify elements that may not be well understood by respondents and problems that may be encountered during the main study. The piloting will be carried out among randomly selected 10 children from a community-based HIV programme. The randomization will be carried out among 8- to 10-year-old who are living with HIV. They will be randomly selected as they come into the clinic for their usual appointments and requested to enroll for the pilot study. The piloting will aid in adapting BENCI in terms of modifying item formats that may not be recognized by respondents, eliminating translation bias among other modifications. In order to improve content validity, inter-rater reliability analysis will be carried out where two raters will review the results of the pretest. One rater will administer the tool amongst the pilot sample, while the other rater will review how the tool is administered and the respondent responds. This work will be qualitative in nature trying to identify and refine the items and their relevance within BENCI.

HIV exposed and unexposed children will be considered as comparative groups—and potentially matched on the patient background characteristics (age and gender). The two comparable groups will be of equal sample sizes which will be calculated using a formula cited in Wittes [12].

In this study sample size computation is based on data from earlier studies in Africa, the $\mu_{1} = 184.7 \left( {sd = 63.7} \right)\;{\text{and}}\;\mu_{2} = 200.6 \left( {sd = 68.7} \right)$ which are derived using KABC-2 between HIV exposed and unexposed [13]. Thus, the assumed pooled standard deviation of the mean difference is approximately (sqrt (68.7^2^ + 63.7^2^)/2) = 66.3. Together with a significance level of 5% and a power of 80%, these result in a total sample size of 544 respondents, 272 in each study arm. So, the study will need to enroll 272 HIV exposed children and 272 unexposed children.

Respondents will be recruited using random stratified sampling by sex and age. A list of all respondents aged 8–11 years in the HIV program and 8–11 years in the primary schools according to gender will then be extracted from the children data base in the institutions. The children’s caregivers will then be requested to give consent on behalf of the children in the schools and the HIV program. A list of all the children with parental consent will then be compiled in readiness for data collection day. On data collection day, the respondents who agree to participate, will be shown the room with neuropsychological assessment tools and proceed with the demographic questionnaire and later on the neuropsychological tests. The process will take around 90–120 min per child.

### Data analysis and presentation

Since the data generated by the BENCI will be collected in computerized format, it will automatically be numerically coded with identifiers attached to different respondents and excel sheets will be generated as programmed in the tool. The Kilifi Toolkit subsets data will be manually keyed into Excel sheets after being numerically coded with identifiers that are attached to differentiate the respondents. Descriptive statistics, as well as, frequency distributions will be used to analyze the demographic traits among other characteristics of the HIV uninfected unexposed (control) and HIV infected (experimental) with the aid of SPSS. Intraclass correlation, in the same software, will be used to calculate test–retest reliability. To examine convergent validity, the raw scores of BENCI subtests will be compared to the raw scores of the subtests in Kilifi Toolkit. A confirmatory factor analysis will be used to assess construct validity. The validity indicators will be several alternative fit statistics as recommended by Hu and Bentler [14]: Chi square its degrees of freedom and its significance value (a good fitting model would show a non-significant value); root mean square error of approximation—RMSEA whose cut off is .06 and below; as well as scoring .95 and above in Comparative Fit Index (CFI) and Tucker–Lewis Index (TLI) [14]. Discriminant validity will be assessed by comparing the neurocognitive scores of the control (HIV unexposed uninfected) to the experimental group (HIV infected) as well as checking on the age sensitivity. This will be assessed using Receiver Operating Characteristics (ROC), where area under the curve will indicate the diagnostic accuracy of BENCI i.e. the ability of the BENCI to correctly classify those with neurocognitive deficits from those without. These statistical calculations will be carried out in SPSS and the diagnostic accuracy indicators will be excellent for .90–1; good for .80–.90; fair for .70–.80; poor for .60–.70; as well as, fail for .50–.60 [15]. Descriptive statistics including frequency tables, percentages, histograms and bar graphs will be used to show results, as well as, explain correlations between the variables. A neurocognitive profile curve will be used to show differences in cognitive outcomes between the experimental and control group.

## Study limitations

The study will be conducted in a community setting; hence the findings may not be replicated within a clinical setting.

## Data Availability

There isn’t any data for sharing at the moment as no datasets have been generated or analyzed.

## Abbreviations

LAMICs: low and middle-income countries
PaM-D: Partnerships for Mental Health Development in Sub-Saharan Africa
NIMH: National Institute of Mental Health
BENCI: Computerized Battery for Neuropsychological Evaluation of Children
CFI: Comparative Fit Index
TLI: Tucker–Lewis Index
HIV: human immunodeficiency virus
KABC 2: Kaufman Assessment Battery for Children, version 2
DSM V: Diagnostic and Statistical Manual of Mental Disorders, 5th Edition
RMSEA: root mean square error of approximation
SPSS: Statistical Package for the Social Sciences
